# Associations of the 2018 World Cancer Research Fund/American Institute of Cancer Research (WCRF/AICR) cancer prevention recommendations with stages of colorectal carcinogenesis

**DOI:** 10.1002/cam4.6119

**Published:** 2023-05-22

**Authors:** Ane Sørlie Kværner, Astrid Riseth Andersen, Hege Berg Henriksen, Markus Dines Knudsen, Anne Marte Wetting Johansen, Anette Hjartåker, Siv Kjølsrud Bøhn, Ingvild Paur, Gro Wiedswang, Sigbjørn Smeland, Trine B. Rounge, Rune Blomhoff, Paula Berstad

**Affiliations:** ^1^ Section for Colorectal Cancer Screening Cancer Registry of Norway Oslo Norway; ^2^ Department of Nutrition University of Oslo Oslo Norway; ^3^ Department of Epidemiology Harvard T.H. Chan School of Public Health Boston Massachusetts USA; ^4^ Department of Transplantation, Division of Surgery, Inflammatory Medicine and Transplantation Oslo University Hospital Oslo Norway; ^5^ Faculty of Chemistry, Biotechnology and Food Science Norwegian University of Life Sciences (NMBU) Ås Norway; ^6^ Department of Clinical Services, Division of Cancer Medicine Oslo University Hospital Oslo Norway; ^7^ Norwegian Advisory Unit on Disease‐related Undernutrition Oslo University Hospital Oslo Norway; ^8^ Department of Gastrointestinal and Children Surgery, Division of Surgery, Inflammatory Medicine and Transplantation Oslo University Hospital Oslo Norway; ^9^ Division of Cancer Medicine Oslo University Hospital Oslo Norway; ^10^ Institute of Clinical Medicine University of Oslo Oslo Norway; ^11^ Department of Research Cancer Registry of Norway Oslo Norway; ^12^ Centre for Bioinformatics, Department of Pharmacy University of Oslo Oslo Norway

**Keywords:** advanced colorectal lesions, cancer prevention recommendations, colorectal cancer, colorectal carcinogenesis, diet, lifestyle

## Abstract

**Background:**

While adherence to cancer prevention recommendations is linked to lower risk of colorectal cancer (CRC), few have studied associations across the entire spectrum of colorectal carcinogenesis. Here, we studied the relationship of the standardized 2018 World Cancer Research Fund/American Institute for Cancer Research (WCRF/AICR) Score for cancer prevention recommendations with detection of colorectal lesions in a screening setting. As a secondary objective, we examined to what extent the recommendations were being followed in an external cohort of CRC patients.

**Methods:**

Adherence to the seven‐point 2018 WCRF/AICR Score was measured in screening participants receiving a positive fecal immunochemical test and in CRC patients participating in an intervention study. Dietary intake, body fatness and physical activity were assessed using self‐administered questionnaires. Multinomial logistic regression was used to estimate odds ratios (ORs) and 95% confidence intervals (CIs) for screen‐detected lesions.

**Results:**

Of 1486 screening participants, 548 were free from adenomas, 524 had non‐advanced adenomas, 349 had advanced lesions and 65 had CRC. Adherence to the 2018 WCRF/AICR Score was inversely associated with advanced lesions; OR 0.82 (95% CI 0.71, 0.94) per score point, but not with CRC. Of the seven individual components included in the score, alcohol, and BMI seemed to be the most influential. Of the 430 CRC patients included in the external cohort, the greatest potential for lifestyle improvement was seen for the recommendations concerning alcohol and red and processed meat, where 10% and 2% fully adhered, respectively.

**Conclusions:**

Adherence to the 2018 WCRF/AICR Score was associated with lower probability of screen‐detected advanced precancerous lesions, but not CRC. Although some components of the score seemed to be more influential than others (i.e., alcohol and BMI), taking a holistic approach to cancer prevention is likely the best way to prevent the occurrence of precancerous colorectal lesions.

## Graphical abstract

While several studies have documented an association between adherence to cancer prevention recommendations and CRC, data are sparse when it comes to the precancerous lesions. In this study, including participants representing the entire spectrum of colorectal carcinogenesis, strong inverse associations were observed between adherence and advanced precancerous lesions, highlighting the importance of adopting a healthy lifestyle early on to prevent the development of CRC. The separate assessment among CRC patients reveals a potential for improving adherence to the recommendations, especially when it comes to limiting alcohol intake and red and processed meat.

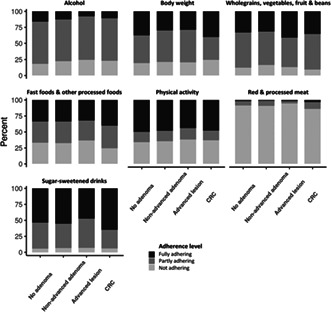

## 1 INTRODUCTION

Globally, colorectal cancer (CRC) is the third most diagnosed cancer in women and men, accounting for over 1.9 million incident cases and 900.000 deaths in 2020.^1^ It has been estimated that about half of all CRC cases could have been avoided by following a healthy diet, being physically active and maintaining a healthy body weight.^2^ In 2018, the World Cancer Research Fund (WCRF) and American Institute for Cancer Research (AICR) issued an expert report (3rd edition since 1997) summarizing the evidence on risk and preventing factors of CRC and other common cancers.^2^ The report concluded with a list of 10 recommendations concerning body weight, physical activity, diet, and breastfeeding aiming at reducing cancer risk and improve overall cancer survival. A standardized scoring system (“the 2018 WCRF/AICR Score”) has been developed to measure adherence to 8 of these 10 recommendations.^3^, ^4^ While there is strong evidence that adherence to select components of the WCRF/AICR Score (e.g., maintaining a healthy body weight and being physically active) protect against CRC,^2^ few studies have examined the joint effect of these on colorectal carcinogenesis, especially for early stage disease development. To the best of our knowledge, no previous study has examined adherence to the 2018 WCRF/AICR Score across the entire spectrum of colorectal carcinogenesis. We therefore investigated the associations of adherence to the standardized 2018 WCRF/AICR Score (excluding the component on breast feeding) with occurrence of colorectal lesions at various stages of the carcinogenic process. We also examined the relative importance of each component of the score for the observed associations. As a secondary objective, we studied to what extent the recommendations were followed in an external cohort of CRC patients.

## 2 METHODS

### 2.1 The BCSN and the CRCbiome study

The CRCbiome study is a prospective cohort sub study within the Bowel Cancer Screening in Norway (BCSN) trial,^5^ a randomized trial comparing once‐only sigmoidoscopy with fecal immunochemical tests (FIT) every 2 years for a maximum of four rounds. The BCSN was initiated in 2012, with follow‐up FIT rounds scheduled to be completed in 2024. Women and men aged 50–74 years at study start, living in two geographic areas in South‐East Norway, were invited to participate. Out of 77,371 individuals invited to FIT screening, 47,432 participated during at least one of the first three screening rounds. This resulted in a participation rate in the FIT screening of 61%. Out of these individuals, those receiving a positive FIT test (i.e., hemoglobin >15 mcg/g feces) during 2017–2021, were eligible for the CRCbiome study. Participants were invited to the CRCbiome study after being informed about their test result, but before attending follow‐up colonoscopy at their local screening center (in Moss or Bærum hospitals). With the invitation letter, participants received two questionnaires to be completed prior to colonoscopy: a lifestyle‐ and demographics questionnaire (LDQ) and a food frequency questionnaire (FFQ). Returning at least one of the questionnaires was regarded as consent to the study. Of 2698 invited, 1653 (61%) agreed to participate. A more detailed description of the CRCbiome study can be found elsewhere.^6^

The BCSN and the CRCbiome study have been approved by the Regional Committee for Medical Research Ethics in South East Norway (Approval no. 2011/1272 and 63148, respectively). The BCSN is also registered at clinicaltrials.gov (National clinical trial (NCT) no. 01538550).

### 2.2 The CRC‐NORDIET study

Participants in the CRC‐NORDIET study composed the external cohort of CRC patients in the present study. CRC‐NORDIET is a randomized controlled trial with two parallel study arms (only data collected prior to intervention start were used in the current study). The overall aim is to investigate the effect of a diet in accordance with the Norwegian food based dietary guidelines on disease‐free survival and overall survival among Norwegian CRC patients.^7^ The participants were recruited between 2012 and 2020. Women and men aged 50–80 years diagnosed with primary invasive CRC at Akershus or Oslo University Hospital were eligible for the study. The cancer needed to be an established primary adenocarcinoma in the colon or the rectum and classified by the ICD‐codes C18‐20, with TNM stages I–III. Participants were invited at the hospitals prior to surgery or by telephone after surgery, and an informed consent needed to be signed before randomization to either the intervention group or the control group. With the invitation letter, participants received a FFQ to be completed prior to intervention start. Of 621 participants signing the consent, 503 (81%) were eligible for the study and participated at baseline, scheduled 2–9 months after surgery. The CRC‐NORDIET study has been approved by the Regional Committee for Medical Research Ethics in South East Norway (Approval no. 2011/836). It is also registered at clinicaltrials.gov (NCT no. 01570010).

### 2.3 Study sample

**FIGURE 1:**
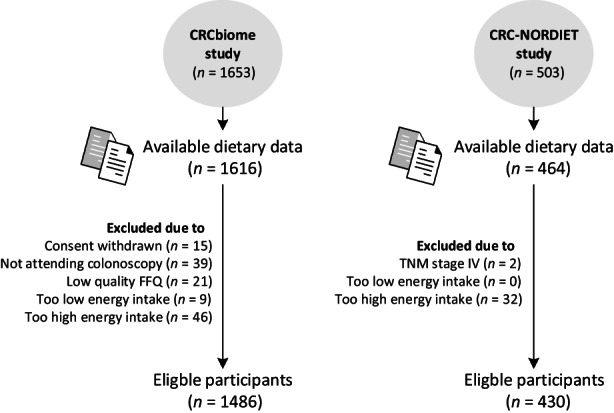
Flowchart of the study participants in CRCbiome and CRC‐NORDIET.

To address the research questions of the present study, the aforementioned study populations CRCbiome and CRC‐NORDIET were included; CRCbiome was used to examine associations between adherence to the 2018 WCRF/AICR Score and screen‐detected colorectal lesions (main objective). CRC‐NORDIET was used to describe adherence in an independent cohort of CRC patients (secondary objective). In CRCbiome, all participants with available dietary information by October 2021 was included (*n* = 1616). Exclusion criteria included non‐attendance on the follow‐up colonoscopy (*n* = 39), withdrawal from the study after inclusion (*n* = 15), delivery of a poor quality FFQ (*n* = 21) or reporting a too low (<600 and <800 kcal/day for women and men, respectively, *n* = 9) or too high (>3500 and >4200 kcal/day for women and men, respectively, *n* = 46) energy intake (Figure 1). This resulted in a study population of 1486 participants; 548 were free from any adenomas, 524 had one or more non‐advanced adenomas, 394 had one or more advanced lesions and 65 had CRC. In CRC‐NORDIET, 464 had available dietary data. Exclusion criteria included presence of a metastatic disease (*n* = 2) or reporting a too low (<600 and < 800 kcal/day for women and men, respectively, *n* = 0) or too high (>3500 and > 4200 kcal/day for women and men, respectively, *n* = 32) energy intake. This resulted in a study population of 430 participants.

### 2.4 Assessment of dietary intake, body fatness, and physical activity

In both studies, dietary data were obtained using self‐administered semiquantitative FFQs, designed to capture the habitual diet the preceding year. The two questionnaires are modified versions of an FFQ developed by the Department of Nutrition, University of Oslo, which has been validated for a variety of nutrients and food groups.^8^, ^9^, ^10^ The version used in CRCbiome had 23 main questions covering 256 food items and an open field for entries not covered by the questionnaire. The CRC‐NORDIET version had 24 main questions covering 282 food items and an open field for entries not covered by the questionnaire. For each food item, participants were asked to record frequency of consumption, ranging from never/seldom to several times a day, and/or amount, typically as portion size given in various household units. Dietary intake was calculated using the food and nutrient calculation system, “kostberegningssystem” (KBS), and the associated database AE‐18, developed at the Department of Nutrition, University of Oslo. AE‐18 is an extended version of the official Norwegian Food Composition Table.^11^ Prior to analyses, all questionnaires were reviewed and evaluated by trained personnel according to “Tutorial for scanning of FFQs and food diaries” prepared by the Department of Nutrition, University of Oslo.

Body mass index was calculated from self‐reported body height and weight in the FFQs. Physical activity level was covered by questions on total number of weekly hours of activity on three different intensity levels in the LDQ in the CRCbiome study. In CRC‐NORDIET, data on physical activity was obtained from a short semi‐quantitative validated FFQ called NORDIET‐FFQ.^12^, ^13^

### 2.5 Operationalization of the 2018 WCRF/AICR Score

The 2018 WCRF/AICR recommendations for cancer prevention were operationalized following a standardized scoring system developed by Shams‐White et al. in 2019.^3^, ^4^ Eight of the 10 cancer prevention recommendations were included: (1) be a healthy weight, (2) be physically active, (3) consume a diet rich in wholegrains, vegetables, fruit, and beans, (4) limit consumption of “fast foods” and other processed foods high in fat, starches, or sugars, (5) limit consumption of red and processed meat, (6) limit consumption of sugar sweetened drinks, (7) limit alcohol consumption and (8) for mothers, breastfeed your baby, if you can (optional). The two remaining recommendations, that is, ‘do not use supplements’ and ‘after a cancer diagnosis, follow our recommendations, if you can’ were left out due to operational redundancy. In the present study, all recommendations were included, except the one concerning breastfeeding due to lack of data. For each recommendation, participants could earn 1, 0.5, or 0 points for fully, partially and not meeting the recommendation, respectively. The total score therefore ranged from 0 to 7 points, higher scores indicating greater adherence to the recommendations. To promote transparency and reproducibility, a detailed overview of how each recommendation was operationalized is given in Table S1, as encouraged by the developers of the score.^4^

### 2.6 Assessment of covariates

Demographic data (i.e., level of education, working status, nationality, and marital status) and information on smoking status were retrieved from the LDQ in the CRCbiome study. The LDQ is a self‐administered, four‐page questionnaire which was piloted in a targeted population prior to study start and adjusted according to participants' feedback. In the CRC‐NORDIET study, demography variables were collected from a self‐administered, one‐page questionnaire. Smoking status was reported in the previously mentioned FFQ. Further information on collection procedures in the two studies can be found elsewhere.^6^, ^7^

### 2.7 Outcome assessment

In the CRCbiome study, the follow‐up colonoscopy formed the basis for the outcome classification. Clinicopathological characteristics of detected lesions were registered by the responsible gastroenterologist using a structured recording system. Based on available clinicopathological information, participants were categorized into the following diagnostic groups: No adenoma, non‐advanced adenoma, advanced lesions, and CRC (any adenocarcinoma of the colon and rectum, i.e., ICD‐10 codes C18‐20). Advanced lesions included both advanced adenomas (any adenoma with villous histology, high‐grade dysplasia, or polyp size greater than or equal to 10 mm) and advanced serrated lesions (any serrated lesion with size ≥10 mm or dysplasia).^14^ In cases of multiple findings, the most severe finding was selected.

In the CRC‐NORDIET study–where all participants were recruited based on their cancer diagnosis, tumor characteristics, including disease severity and localization, were retrieved from electronic patient records.

### 2.8 Statistical analyses

Descriptive statistics are given as median (p25, p75) and numbers (percentages) for continuous and categorical variables, respectively.

Multinomial logistic regression analyses were used to calculate the odds ratios (ORs) and 95% confidence intervals (CIs) for screen‐detected colorectal lesions (presence of non‐advanced adenoma, advanced lesions, and CRC) relative to no adenoma by adherence to the 2018 WCRF/AICR Score. Participants were divided into quartiles based on level of adherence to the 2018 WCRF/AICR Score: Q1; ≤2.75 points (reference category), Q2; 3.0–3.5 points, Q3; 3.75–4.25 points, and Q4; ≥4.5 points. The analyses were conducted in the screening population as a whole and stratified by sex. A separate analysis was also conducted by precursor lesion type (i.e., advanced adenoma or advanced serrated lesion relative to no adenoma). To examine the relative importance of each component of the score for the observed associations, seven additional scores were created, each subtracting a different component of the score. The relationships of these modified scores with colorectal lesions were then examined. Effect estimates were calculated for one point increase in the respective scores, with the original score included for comparison purposes. In addition to the assessment of adherence to the 2018 WCRF/AICR Score (and the modified ones), the individual diet and lifestyle recommendations were examined separately. For these analyses, ORs and 95% CIs by one point increase in the components (i.e., going from not adhering to fully adhering to the recommendation) were calculated.

All association analyses were adjusted for the following covariates: age (continuous), sex, energy intake (continuous), smoking status (current smoker, past smoker, non‐smoker, missing), education level (primary school, high school, collage/university, missing), and family history of CRC (yes, no, and unknown). The covariate selection was based on a priori knowledge on the relationship between diet and lifestyle and colorectal carcinogenesis.^2^, ^15^, ^16^

In line with the most recent statement from the American Statistical Association on *p*‐values,^17^ emphasis was put on effect sizes, variation, and uncertainty of the data rather than *p*‐values in the interpretation of the results. All statistical analyses were performed using RStudio, version 3.6.3 (The R Foundation for Statistical Computing). The main R packages used included those within the Tidyverse,^18^ as well as skimr, nnet and vgam.

## 3 RESULTS

### 3.1 Key characteristics of the study population

Characteristics of the study population by study and stage of the carcinogenic process are presented in Table 1. Among the CRCbiome participants, the median age was 67 years, ranging from 66 to 68 years across the diagnostic groups. For all carcinogenic stages, there was a dominance of male participants (55%–63%). The majority of participants (>90%) were recruited prior to becoming aware of a potential clinical finding. In the external cohort of CRC patients CRC‐NORDIET, the median age of participants was 67 years, 54% being male. Participants were either recruited in relation to the hospital admission (25%) or in the recovery period following surgery (75%).

**TABLE 1:** Key characteristics of the study population by study and disease stage (*n* = 1916).^a^

	CRCbiome (*n* = 1486)				CRC‐NORDIET (*n* = 430)
Variables	No adenoma (*n* = 548)	Non‐advanced adenoma (*n* = 524)	Advanced lesion (*n* = 349)	CRC (*n* = 65)	CRC (*n* = 430)
Demography and lifestyle					
Age, years	65.8 (60.7, 71.0)	67.8 (62.8, 72.5)	67.7 (62.5, 72.0)	68.0 (62.6, 72.8)	67.0 (60.0, 72.0)
Male sex, *n* (%)	267 (48.7)	305 (58.2)	218 (62.5)	36 (55.4)	232 (54.0)
Nationality, *n* (%)					
Native	501 (91.4)	471 (89.9)	316 (90.5)	57 (87.7)	303 (70.5)
Non‐native	34 (6.2)	27 (5.1)	16 (4.6)	5 (7.7)	17 (4.0)
Missing	13 (2.4)	26 (5.0)	17 (4.9)	3 (4.6)	110 (25.6)
Family history of CRC, *n* (%)					
Yes	84 (15.3)	89 (17.0)	65 (18.6)	17 (26.2)	65 (15.1)
No	407 (74.3)	390 (74.4)	252 (72.2)	43 (66.2)	229 (53.3)
Unknown	57 (10.4)	45 (8.6)	32 (9.2)	5 (7.7)	136 (31.6)
Education, *n* (%)					
Primary school	93 (17.0)	91 (17.4)	58 (16.6)	9 (13.8)	42 (9.8)
High school	223 (40.7)	195 (37.2)	134 (38.4)	28 (43.1)	177 (41.2)
University/college	225 (41.1)	231 (44.1)	147 (42.1)	28 (43.1)	202 (47.0)
Missing	7 (1.3)	7 (1.3)	10 (2.9)	0 (0.0)	9 (2.1)
Marital status, *n* (%)					
Married/cohabiting	454 (82.8)	399 (76.1)	267 (76.5)	51 (78.5)	305 (70.9)
Not married/non‐cohabiting	89 (16.2)	119 (22.7)	72 (20.6)	14 (21.5)	116 (27.0)
Missing	5 (0.9)	6 (1.1)	10 (2.9)	0 (0.0)	9 (2.1)
Working status, *n* (%)					
Employed	199 (36.3)	171 (32.6)	109 (31.2)	19 (29.2)	121 (28.1)
Retired/unemployed	343 (62.6)	347 (66.2)	230 (65.9)	46 (70.8)	295 (68.6)
Missing	6 (1.1)	6 (1.1)	10 (2.9)	0 (0.0)	14 (3.3)
Smoking status, *n* (%)					
Current smoker	63 (11.5)	86 (16.4)	59 (16.9)	6 (9.2)	44 (10.2)
Non smoker	478 (87.2)	430 (82.1)	281 (80.5)	59 (90.8)	385 (89.5)
Missing	7 (1.3)	8 (1.5)	9 (2.6)	0 (0.0)	1 (0.2)
**Clinical information**					
Hospital, *n* (%)					
Bærum	237 (43.2)	261 (49.8)	179 (51.3)	35 (53.8)	–
Moss	311 (56.8)	263 (50.2)	170 (48.7)	30 (46.2)	–
Ullevål	–	–	–	–	221 (51.4)
Akershus	–	–	–	–	209 (48.6)
Tumor localization^b^, *n* (%)					
Colon	–	–	–	32 (49.2)	251 (58.4)
Rectum	–	–	–	33 (50.8)	174 (40.5)
Missing	–	–	–	0 (0.0)	5 (1.2)
TNM stage, *n* (%)					
I	–	–	–	36 (55.4)	115 (26.7)
II	–	–	–	17 (26.2)	143 (33.3)
III	–	–	–	10 (15.4)	127 (29.5)
IV				2 (3.1)	–
Missing	–	–	–	0 (0.0)	45 (10.5)
**Completion of the FFQ**					
Diagnosis known, *n* (%)					
Yes	31 (5.7)	56 (10.7)	36 (10.3)	3 (4.6)	430 (100)
No	517 (94.3)	468 (89.3)	313 (89.7)	62 (95.4)	0 (0.0)
Time relative to lesion removal, days	−7 (−13, −2)	−5.5 (−13, −1)	−5 (−14, −1)	−8 (−14, −3)	86 (−0.5, 127)

^a^ Values are median (p25, p75) for continuous variables and *n* (%) for categorical variables.

^b^ One of the colon cancer cases in CRCbiome was also diagnosed with a primary invasive rectum cancer.

Abbreviations: CRC, colorectal cancer, FFQ, food frequency questionnaire, TNM, tumor, node, metastasis.

### 3.2 Adherence to the 2018 WCRF/AICR Score

Descriptive statistics of the 2018 WCRF/AICR Score, as well as the individual diet and lifestyle components forming the basis for the score, is provided in Table 2 and Table S**2**. Only data for the CRCbiome participants are presented. The median (p25, p75) adherence to the recommendations was 3.5 (2.8, 4.3) points, ranging from 3.5 to 3.8 points across the diagnostic groups (Table 2). None of the CRCbiome participants adhered to all recommendations, the highest score being 6.5, achieved by 3 (0.2%) participants. Women scored slightly higher than men; 3.8 (3.0, 4.5) vs. 3.5 (2.8, 4.0) points, respectively (Table S2). For selected dietary components (i.e., fiber and red and processed meat), moderate correlations with energy intake was observed (Table S2).

**TABLE 2:** Summary of diet and lifestyle characteristics of the 2018 WCRF/AICR Score by disease stage. Only CRCbiome participants are included (*n* = 1486). Numbers are median (p25, p75).^a^

	No adenoma (*n* = 548)	Non‐advanced adenoma (*n* = 524)	Advanced lesion (*n* = 349)	CRC (*n* = 65)
**Global scoring**				
WCRF/AICR Score, points	3.5 (3.0, 4.5)	3.5 (2.8, 4.3)	3.5 (2.8, 4.0)	3.8 (3.0, 4.5)
**Individual recommendations**				
*Be a healthy weight*				
BMI, kg/m^2^	26.3 (23.7, 28.9)	26.6 (24.4, 29.4)	26.6 (24.5, 29.3)	25.5 (23.7, 29.7)
*Be physically active*				
Moderate‐vigorous physical activity, min/week	180 (8, 315)	135 (0, 300)	135 (0, 304)	135 (15, 300)
*Eat whole grains, vegetables, fruits, and beans*				
Fruits and vegetables, g/day	422 (283, 597)	401 (257, 579)	439 (274, 610)	518 (307, 644)
Fiber, g/day	27.6 (21.8, 34.8)	27.0 (21.1, 34.5)	29 (22.5, 36.7)	26.0 (21.5, 35.7)
*Limit fast foods and processed foods*				
NOVA‐classified aUPFs^b^, E%	16.4 (11.3, 21.5)	16.1 (11.6, 21.4)	16.9 (12.0, 22.0)	15.1 (9.13, 19.4)
*Limit red and processed meat*				
Red meat, g/day	69.1 (44.2, 95.8)	68.4 (47.7, 96.4)	75.6 (51.7, 113.0)	61 (38.8, 88.6)
Processed meat, g/day	46.3 (29.7, 68.5)	46.7 (29.5, 67.5)	52.6 (34.3, 76.6)	41.2 (25.3, 58.4)
*Limit sugar‐sweetened drinks*				
Sugar sweetened drinks, g/day	0 (0, 42)	0 (0, 49)	14 (0, 42)	0 (0, 35)
*Limit alcohol*				
Alcohol, g/day	7.4 (1.8, 17.5)	9.3 (2.0, 19.6)	11.0 (3.5, 21.0)	8.8 (3.2, 21.7)

^a^ For continuous variables, numbers may vary due to missing information.

^b^ The aUPF variable was created based on the NOVA classification system. Food items already included in other components of the score (e.g., sugar‐sweetened drinks and red and processed meats) were left out to avoid double penalization.

Abbreviations: AICR; American Institute for Cancer Research, aUPFs; adapted ultra‐processed foods, p; percentile, WCRF; World Cancer Research Fund.

**FIGURE 2:**
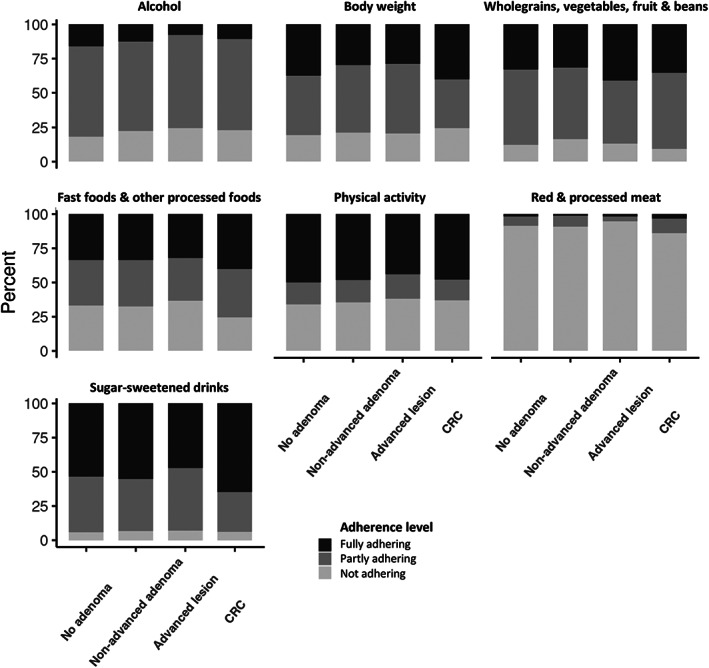
Proportion of participants who fully, partly and do not adhere to the individual Cancer Prevention Recommendations from WCRF/AICR of 2018 by stage of the carcinogenic process. Only the CRCbiome participants are included (*n* = 1486).

Adherence to the individual diet and lifestyle recommendations (i.e., proportion fully, partly, and not adhering to the recommendations) is presented in Figure 2. Although the level of adherence varied by diagnostic group, there were some general patterns. In general, highest adherence was seen for the recommendations of limiting the amounts of sugar‐sweetened beverages (47%–65% fully adhering) and being physically active (44%–50% fully adhering). For the recommendations on having a healthy body weight, eating a diet rich in wholegrains, vegetables, fruit, and beans and limiting the consumption of “fast foods” and other processed foods high in fat, starches, and sugars, approximately one third of the participants fully adhered to the recommendations. The lowest adherence to the recommendations were seen for limiting alcohol intake and consumption of red and processed meat, where 8%–16% and 1%–3% fully adhered to the recommendation, respectively.

### 3.3 The 2018 WCRF/AICR Score and stages of the carcinogenic process

Associations between adherence to the 2018 WCRF/AICR Score and presence of screen‐detected lesions are shown in Table 3. Compared to those having the lowest adherence to the WCRF/AICR Score (Q1, ≤2.75 points), participants achieving higher scores had a reduced probability of advanced precursor lesions (*p*_trend_ of 0.012). Compared to those having the lowest adherence, participants in the fourth quartile (≥4.5 points) had an OR (95% CI) for advanced lesions of 0.66 (0.44, 0.98). Per each point increase in the score, the probability of advanced lesions was lowered by 18% (OR (95% CI) of 0.82 (0.71, 0.94), Table 4). The inverse association was present in both sexes, although only significant in men (*p*_trend_ of 0.031 vs. 0.25 for women, Table 3). In a supplementary analysis of adherence by precancer lesion subtype, higher scores tended to be inversely associated with both advanced adenoma and advanced serrated lesion (Figure S1). For CRC, no associations were observed.

**TABLE 3:** Odds ratios (ORs) and 95% confidence intervals (CIs) for presence of non‐advanced adenoma, advanced lesions and CRC relative to no adenoma by level of adherence to the 2018 WCRF/AICR Cancer Prevention Recommendations. Only CRCbiome participants are included in the analyses (*n* = 1486).^a^

	Stage of the carcinogenic process						
	No adenoma (*n* = 548)	Non‐advanced adenomas (*n* = 524)		Advanced lesions (*n* = 349)		CRC (n = 65)	
	*n*	*n*	OR (95% CI)	*n*	OR (95% CI)	*n*	OR (95% CI)
Overall							
Q1	134	143	Ref.	108	Ref.	12	Ref.
Q2	142	132	0.90 (0.64, 1.26)	104	0.91 (0.63, 1.31)	18	1.38 (0.64, 3.00)
Q3	123	134	0.99 (0.70, 1.41)	64	**0.63 (0.42, 0.94)**	17	1.42 (0.64, 3.15)
Q4	149	115	0.75 (0.53, 1.08)	73	**0.66 (0.44, 0.98)**	18	1.28 (0.57, 2.85)
*p*_trend_			0.21		**0.012**		0.60
Men							
Q1	75	98	Ref.	74	Ref.	6	Ref.
Q2	77	77	0.74 (0.47, 1.16)	73	0.90 (0.57, 1.45)	9	1.43 (0.48, 4.27)
Q3	64	77	0.83 (0.51, 1.33)	39	**0.56 (0.33, 0.96)**	12	2.19 (0.75, 6.37)
Q4	51	53	0.73 (0.44, 1.22)	32	0.63 (0.35, 1.11)	6	1.93 (0.63, 5.90)
*p*_trend_			0.28		**0.031**		0.18
Women							
Q1	59	45	Ref.	34	Ref.	6	Ref.
Q2	65	55	1.18 (0.69, 2.02)	31	0.82 (0.45, 1.52)	9	1.22 (0.40, 3.71)
Q3	59	57	1.31 (0.76, 2.28)	25	0.69 (0.36, 1.33)	5	0.68 (0.19, 2.41)
Q4	98	62	0.89 (0.53, 1.52)	41	0.72 (0.40, 1.30)	9	0.72 (0.23, 2.26)
*p*_trend_			0.66		0.25		0.39

^a^ Odds ratios (ORs) and 95% confidence intervals (CIs) are obtained from multinomial logistic regression analyses adjusting for the following covariates: age (continuous), sex (except in the sex‐specific analyses), energy intake (continuous), smoking status (current smoker, past smoker, non‐smoker, missing), education level (primary school, high school, collage/university, missing) and family history of CRC (yes, no and unknown).

**TABLE 4:** Odds ratios (ORs) and 95% confidence intervals (CIs) for presence of non‐advanced adenoma, advanced lesions and CRC relative to no adenoma by one point increase in the 2018 WCRF/AICR Score overall and after removing each component of the score. Only CRCbiome participants are included in the analyses (*n* = 1486).^a^

	Non‐advanced adenoma (*n* = 524)	Advanced lesion (*n* = 349)	CRC (*n* = 65)
WCRF/AICR Score	0.89 (0.78, 1.01)	**0.82 (0.71, 0.94)**	1.08 (0.82, 1.41)
WCRF/AICR Score—BMI	0.94 (0.81, 1.08)	**0.84 (0.72, 0.99)**	1.14 (0.84, 1.53)
WCRF/AICR Score—Physical activity	**0.84 (0.72, 0.99)**	**0.79 (0.66, 0.94)**	1.18 (0.85, 1.64)
WCRF/AICR Score—Fruit, vegetables and fiber	0.88 (0.77, 1.02)	**0.78 (0.66, 0.91)**	1.04 (0.77, 1.40)
WCRF/AICR Score–Fast foods and ultra processed foods	**0.86 (0.74, 0.99)**	**0.77 (0.65, 0.91)**	1.02 (0.74, 1.40)
WCRF/AICR Score—Red and processed meats	0.88 (0.77, 1.00)	**0.81 (0.70, 0.94)**	1.04 (0.78, 1.38)
WCRF/AICR Score–Sugar‐sweetened beverages	**0.86 (0.75, 0.99)**	**0.80 (0.69, 0.94)**	1.03 (0.77, 1.38)
WCRF/AICR Score—Alcohol	0.92 (0.81, 1.05)	0.87 (0.75, 1.00)	1.15 (0.87, 1.52)

^a^ Odds ratios (ORs) and 95% confidence intervals (CIs) are obtained from multinomial logistic regression analyses adjusting for the following covariates: age (continuous), sex, energy intake (continuous), smoking status (current smoker, past smoker, non‐smoker, missing), education level (primary school, high school, collage/university, missing) and family history of CRC (yes, no and unknown).

Abbreviations: AICR, American Institute for Cancer Research, CI; confidence interval, OR; odds ratio, WCRF; World Cancer Research Fund.

### 3.4 Relative importance of the individual recommendations for the observed associations

To study the relative importance of each component of the score for presence of screen‐detected lesions, seven new scores were created, each subtracting a different component of the score (Table 4). The inverse association observed between adherence to the WCRF/AICR Score and advanced precancerous lesions remained significant for all modifications of the score, except when subtracting the alcohol component, where a borderline significant association was observed. Based on the change in effect estimates, the subtraction of alcohol and BMI from the score seemed to be most influential, both resulting in a weakening of the relationship (from 18% lower probability to 13 and 16%, respectively). The importance of alcohol and BMI for the observed associations was confirmed in a supplementary analysis examining the relationship of each recommendation with presence of precancerous lesions (Figure S2). Going from not adhering to fully adhering to the recommendations concerning alcohol and BMI, respectively, resulted in ORs (95% CIs) for advanced lesions of 0.47 (0.29, 0.77) and 0.61 (0.41, 0.91). For the other recommendations, no associations with precancerous lesions were detected.

### 3.5 Adherence to the 2018 WCRF/AICR Score in the external cohort of CRC patients

**FIGURE 3:**
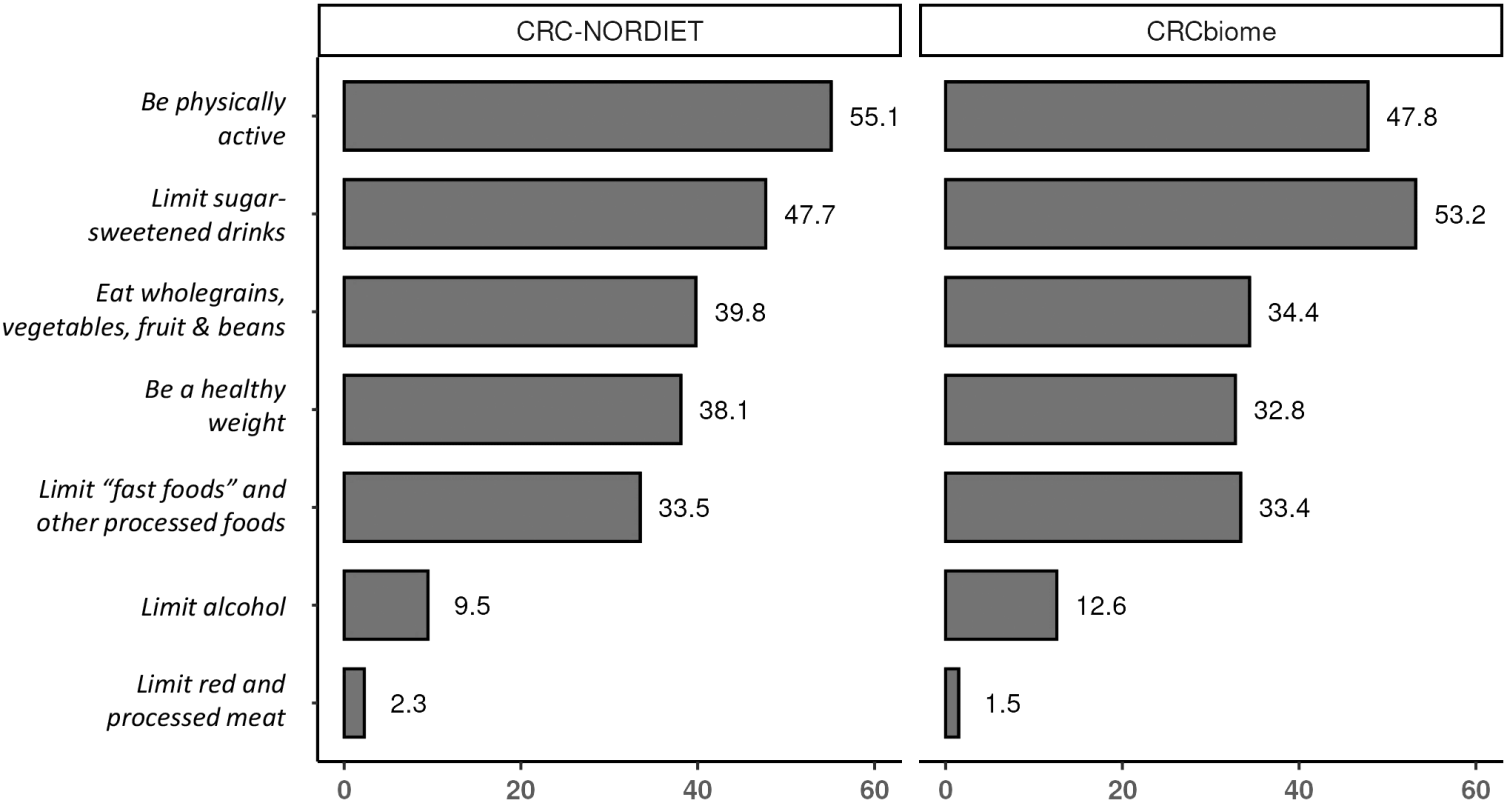
Proportion of the CRC patients in CRC‐NORDIET (*n* = 430) and CRCbiome (*n* = 65) who fully adhere to the individual Cancer Prevention Recommendations from WCRF/AICR of 2018.

To examine to what extent the 2018 WCRF/AICR Cancer Prevention Recommendations were being followed by patients already diagnosed with CRC, the intervention study CRC‐NORDIET was included. The proportion of CRC patients who fully adhered to each recommendation was calculated and visualized in descending order with the numbers for the screen‐detected CRCs included for comparison (Figure 3). In line with what was observed for the CRCbiome population as a whole (Figure 2), the highest adherence was seen for the recommendations on being physically active and limiting the intake of sugar‐sweetened beverages (55% and 48% fully adhering, respectively). Thereafter followed the recommendations on eating wholegrains, vegetables, fruit and beans, having a healthy body weight, and limiting the amount of “fast foods” and other processed foods, all having adherence levels between 30% and 40%. The lowest adherence was seen for the recommendations concerning limiting the intake of alcohol and the amount of red and processed meat consumed, where only 10% and 2%, adhered, respectively. The ordering of adherence levels for the individual components were more or less similar to that of the CRC patients in CRCbiome.

## 4 DISCUSSION

In this large cross‐sectional investigation among screening participants, adhering to the 2018 WCRF/AICR Score was inversely associated with presence of advanced precancerous lesions, but not CRC. Of the seven diet and lifestyle recommendations making up the score, the recommendations on limiting alcohol intake and having a healthy body weight seemed to be most influential.

Various studies have investigated the association of adherence to either the 2007^19^, ^20^, ^21^ or 2018^22^, ^23^, ^24^, ^25^ edition of the cancer prevention recommendations from WCRF/AICR and risk of CRC. However, to the best of our knowledge, no study before the present has assessed the associations across the entire spectrum of colorectal carcinogenesis. Using this approach, we demonstrate that adherence to the 2018 WCRF/AICR cancer prevention recommendations was strongly inversely associated with detection of precancerous lesions, in particular the high‐risk lesions, in a dose–response manner. The lowest probability of lesion detection was seen among those adhering to just above half (≥4.5/7 points) or more of the recommendations. The inverse associations were observed irrespective of histopathological subtype (although not reaching statistical significance) and among both sexes (although only significant in men). In a comparable study, Erben et al.^26^ investigated the associations of a healthy diet and lifestyle score with colorectal lesions, also representing the entire spectrum of CRC development. In that cross‐sectional investigation, including more than 13,000 German screening participants, strong inverse associations were observed with the presence of hyperplastic polyps, non‐advanced adenomas, and advanced colorectal neoplasms, the latter group consisting mostly of advanced adenomas (>90%). Inverse associations between a healthy lifestyle pattern and precancerous colorectal lesions have also been observed in the main BCSN pilot population.^27^, ^28^, ^29^ Together, these findings support the importance of adhering to diet and lifestyle recommendations to prevent early‐stage colorectal carcinogenesis.

In the present study, adherence to the 2018 WCRF/AICR Score was not associated with detection of CRC at colonoscopy. There are several potential reasons for this lack of association. First, the cross‐sectional design makes the study prone to reverse causality. It is possible that the progression of a gastrointestinal tumor prior to screening may have led to involuntary changes in diet or lifestyle of relevance to the score (e.g., a reduction in BMI due to disease‐related malnutrition^30^). In support of this, we have previously shown that a substantial fraction (44%) of the screen‐detected CRCs in the BCSN trial present with some kind of bowel symptom at colonoscopy.^31^ In particular, the presence of rectal bleeding, changes in bowel habits, and abdominal pain have been linked to detection of CRC, all of which may influence nutritional status. Second, the relatively low number of screen‐detected CRCs compared to the other lesions studied (65 with CRC compared to 524 and 349 with non‐advanced and advanced lesions, respectively) reduced statistical power, increasing the possibility of a Type I error.

Of the seven cancer prevention recommendations, being compliant with the recommendations on limiting alcohol intake and having a healthy body weight seemed to be most important for lowering the probability of precancerous lesion detection. That higher alcohol intake and increased body weight are linked to carcinogenic development is supported by meta‐analyses on adenoma risk,^32^, ^33^ as well as the latest expert report from WCRF/AICR on CRC.^2^ In separate investigations of the Global Burden of Disease Project, focusing on the cancer burden attributed to harmful alcohol intake and body weight measures, respectively, it was estimated that as many as 8%–9% and 6%–7% of the incident colon and rectal cancer cases could have been avoided by the elimination of these risk factors.^34^, ^35^ Together with the recommendation on limiting red and processed meat intake, these were also the ones where adherence were the lowest, irrespective of carcinogenic stage studied (also including the external cohort of CRC patients). Increasing the public's awareness of the importance of adhering to the alcohol and body weight recommendations is likely of great importance to lower the cancer burden attributable to these risk factors.

Except for adherence to the alcohol and body weight recommendations, no associations between the other cancer prevention recommendations and presence of precancerous lesions were observed. Given the strong inverse association found for the score as a whole, we speculate that adhering to multiple recommendations in combination—as an integrated package of lifestyle behaviors–is more important for CRC prevention than adherence to each and every factor alone. Indeed, the importance of taking a holistic approach to cancer prevention represents one of the major shifts in focus in the cancer prevention recommendations of 2018 compared to earlier versions.

It is also possible that there are better ways of operationalizing the recommendations. For instance, we were not able to show an association between adherence to the red and processed meat recommendation and presence of precancerous lesions, although evidence linking these food items to CRC development is considered strong,^2^, ^36^ and also shown in the CRCbiome population.^37^ This could suggest that the cut points to achieve full score, particularly those for processed meat (<3 g/day, fulfilled by only 2% of participants) were unnecessarily strict. A recent comparative analysis of 18 dietary patterns and risk of CRC^25^ suggests a potential for further refining the 2018 WCRF/AICR recommendations by making use of already available dietary patterns (e.g., those reflecting hyperinsulinemia, hypertension, chronic inflammation, and a Western dietary pattern).

A major strength of the present study is the detection of colorectal lesions in a screening setting, leaving the participants unknown of the screening result at time of diet and lifestyle recall. A further strength is the use of a standardized scoring system for measuring adherence to the 2018 WCRF/AICR cancer prevention recommendations, enabling cross‐study comparisons.^3^, ^4^ The access to comprehensive high‐quality data on diet and lifestyle, allowed a thorough evaluation of adherence to each recommendation and the operationalization was carried out by two registered dietitians.

The main limitation of the study is the cross‐sectional design. In addition to the problem of reverse causality, discussed above, only having access to diet and lifestyle information reflecting the last year prior to the colonoscopy represents a limitation. Considering the long latency period of CRC, obtaining this information a minimum of 10–15 years prior to diagnosis would be ideal. Second, the findings could be limited by the use of self‐reported data for constructing the score. However, the questionnaires used have been validated for the majority of components included in the score,^8^, ^9^, ^38^, ^39^, ^40^, ^41^ and mostly shown to produce acceptable results. Last, all participants recruited to CRCbiome were FIT positive. Although this contributed to a higher proportion of precancerous and cancerous lesions, it likely also increased the frequency of other pathologies, making the comparison group less clean. The selective recruitment of FIT positive participants also reduces the generalizability of our findings to the general population.

To conclude, in this high‐risk group of screening participants, reflecting the entire spectrum of colorectal carcinogenesis, high adherence to the 2018 WCRF/AICR cancer prevention recommendations was inversely associated with presence of advanced precancerous lesions, but not CRC. Although select components of the score seemed to be more important for lesion detection than others (i.e., alcohol and BMI), the largest preventive effects could likely be achieved by adhering to multiple cancer prevention recommendations in combination.

## AUTHOR CONTRIBUTIONS

**Ane Sørlie Sørlie Kværner:** Conceptualization (equal); data curation (equal); formal analysis (lead); investigation (equal); methodology (equal); project administration (equal); visualization (lead); writing – original draft (lead); writing – review and editing (lead). **Astrid Riseth Andersen:** Conceptualization (equal); data curation (equal); formal analysis (supporting); investigation (equal); methodology (equal); project administration (equal); visualization (supporting); writing – original draft (supporting); writing – review and editing (supporting). **Hege Berg Henriksen:** Conceptualization (equal); data curation (supporting); formal analysis (supporting); investigation (equal); methodology (equal); project administration (equal); visualization (supporting); writing – review and editing (supporting). **Markus Dines Knudsen:** Writing – review and editing (supporting). **Anne Marte Wetting Johansen:** Data curation (supporting); writing – review and editing (supporting). **Anette Hjartaker:** Writing – review and editing (supporting). **Siv Kjølsrud Bøhn:** Writing – review and editing (supporting). **Ingvild Paur:** Writing – review and editing (supporting). **Gro Wiedswang:** Writing – review and editing (supporting). **Sigbjørn Smeland:** Writing – review and editing (supporting). **Trine Ballestad Rounge:** Funding acquisition (equal); writing – review and editing (supporting). **Rune Blomhoff:** Conceptualization (equal); formal analysis (supporting); funding acquisition (equal); investigation (equal); methodology (equal); project administration (equal); supervision (supporting); visualization (supporting); writing – review and editing (supporting). **Paula Berstad:** Conceptualization (equal); formal analysis (supporting); funding acquisition (equal); investigation (equal); methodology (equal); project administration (equal); supervision (lead); visualization (supporting); writing – review and editing (supporting).

## FUNDING INFORMATION

The CRCbiome study has received funding from the Norwegian Cancer Society (grant nos. 190179 and 198048), the Norwegian Cancer Society's umbrella organization for cancer research (“Kreftforeningens paraplystiftelse for kreftforskning”) and the Research Council of Norway (grant no. 280667). The CRC‐NORDIET study is funded by the Throne Holst Foundation of Nutrition Research, the Norwegian Cancer Society, the Research Council of Norway and the South Eastern Norway Regional Health Authority.

## CONFLICT OF INTEREST STATEMENT

Rune Blomhoff is a shareholder of Vitas, Oslo, Norway. The other authors declare no potential conflicts of interest.

## TRIAL REGISTRATION

ClinicalTrials.gov Identifier: NCT01538550 (Bowel Cancer Screening in Norway (BCSN) trial) and NCT01570010 (CRC‐NORDIET).

## Supporting information


Data S1.

## Data Availability

The data generated in the two studies are not publicly available due to the principles and conditions set out in articles 6 [1] (e) and 9 [2] (j) of the General Data Protection Regulation (GDPR), but are available upon reasonable request from the principal investigators: CRCbiome; Paula Berstad and Trine B. Rounge (crcbiome@kreftregisteret.no), CRC‐NORDIET; Rune Blomhoff (rune.blomhoff@medisin.uio.no).
