# Supplementary material for: Associations of the 2018 World Cancer Research Fund/American Institute of Cancer Research (WCRF/AICR) cancer prevention recommendations with stages of colorectal carcinogenesis
Source: Cancer Med. 2023 May 22;12(13):14806–19. doi: 10.1002/cam4.6119 (PMC10358192; doi:10.1002/cam4.6119)
Supplement: Supplementary file 1 — Data S1. [file CAM4-12-14806-s001.docx]

### **Supplementary Data**

**Supplementary Table 1.** The 2018 WCRF/AICR Cancer Prevention Recommendations with suggested^1^ and applied operationalization.

| **2018 WCRF/AICR Recommendations with suggested operationalization** | | | **Operationalization in the current study** | |
| --- | --- | --- | --- | --- |
| 2018 WCRF/AICR recommendations | Recommend operationalization of recommendations with suggested scoring | Suggested scoring | Applied scoring | Comments and considerations |
| **1. Be a healthy weight** | **BMI (kg/m^2^):**  18.5–24.9  25–29.9  <18.5 or ≥30  **Waist circumference (cm):**  M: <94 / W: <80  M: 94–<102 / W: 80–<88  M: ≥102 / W: ≥88 | 0.5  0.25  0  0.5  0.25  0 | 1.0  0.5  0  -  -  - | Only BMI available for both studies  Calculated from self-reported body weight (kg) and height (m^2^) |
| **2. Be physically active** | **Total moderate-vigorous physical activity (min/wk):**  ≥150  75–<150  <75 | 1  0.5  0 | 1  0.5  0 | Calculated as the sum of self-reported moderate (min/wk) and vigorous (min/wk) physical activity, the latter weighted by a factor of two to best reflect the recommendation |
| **3. Eat a diet rich in wholegrains, vegetables, fruit and beans** | **Fruits and vegetables (g/day):**  ≥400  200–<400  <200  **Total fiber (g/day):**  ≥30  15–<30  <15 (0) | 0.5  0.25  0  0.5  0.25  0 | 0.5  0.25  0  0.5  0.25  0 | Fruits and vegetables included all fresh, frozen and conserved fruits, berries and vegetables.  For the following items, a prespecified proportion was included to account for other ingredients in the product:   - Jam and marmalade (50%) - Vegetable dishes (50%)   The food item ‘vegetable soup’ was left out to lower the chances of overestimation due to the expected high contribution of dried soups.  Juice and juice concentrate were not included. Legumes were also left out. |
| **4. Limit consumption of “fast foods” and other processed foods high in fat, starches or sugars** | **Percent of total kcal from ultra-processed foods (aUPFs):**  Tertile 1  Tertile 2  Tertile 3 | 1  0.5  0 | 1  0.5  0 | The aUPF variable was constructed based on the NOVA classification system(36). Food items already included in other components of the score (e.g. sugar-sweetened drinks and red and processed meats) were left out to avoid double penalization.  The following items were defined as aUPFs: White bread, tortilla, chapatti and related products, sandwich biscuits, breakfast cereals with added sugar, cakes, desserts, ice cream, sorbet, chocolates, sweets, snacks, processed products of milk or cream such as vanilla sauce, honey/syrup (50% included), jam/marmalade and other spreads with added sugar, artificial sweeteners, margarine and mixed products of margarine and butter (50% included), mayonnaise, french fries, mashed potatoes, vegetable soups, vegetable products, fish products, sauces, dry soups, bouillon powder/cubes, artificially sweetened lemonade and soda, milk substitutes and liquor  The aUPF variable was constructed by adding up the listed items in kcal/day and dividing the total by daily caloric intake times hundred.  Tertiles were created separately by study. |
| **5. Limit consumption of red and processed meat** | **Total red meat (g/wk) and processed meat (g/wk):**  Red meat <500 and processed meat <21  Red meat <500 and processed meat 21–<100  Red meat >500 or processed meat ≥100 | 1  0.5  0 | 1  0.5  0 | Red meat included all non-white meat, except wild game meat. Both processed and non-processed red meat were included.  Processed meat included all processed meat products, irrespective of animal origin. |
| **6. Limit consumption of sugar-sweetened drinks** | **Total sugar-sweetened drinks (g/day):**  0  >0–≤250  >250 | 1  0.5  0 | 1  0.5  0 | Sugar-sweetened drinks included lemonade, soda and milk with added sugar, juice concentrate and mixed drinks. |
| **7. Limit alcohol consumption** | **Total ethanol (g/day):**  0  M: >0–≤28 (2 drinks) / W: ≤14 (1 drink)  M: >28 (2 drinks) / W: >14 (1 drink) (0) | 1  0.5  0 | 1  0.5  0 |  |
| **8. For mothers: breastfeed your baby, if you can (optional)** | **Exclusively breastfed over lifetime for a total of:**  6+ months  >0–<6 months  Never | 1  0.5  0 | -  -  - | Data not available |

*^1^Shams-White, et al. Operationalizing the 2018 World Cancer Research Fund/American Institute for Cancer Research (WCRF/AICR) Cancer Prevention Recommendations: A Standardized Scoring System. Nutrients (2019).*

| **Supplementary Table 2.** Summary of diet and lifestyle characteristics of the 2018 WCRF/AICR Score in the study population as a whole (n=1,486) and by sex (men: 826, women: 660)^1^. | | | | |
| --- | --- | --- | --- | --- |
|  | Median  (p25, p75) | | | Correlation with energy^2^ |
|  | **Overall** | **Men** | **Women** |  |
| **Global scoring** |  |  |  |  |
| WCRF/AICR Score, points | 3.5 (2.8, 4.3) | 3.5 (2.8, 4.0) | 3.8 (3.0, 4.5) | 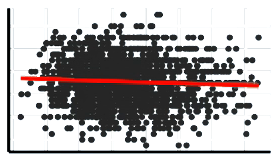 |
| **Individual recommendations** |  |  |  |  |
| **Be a healthy weight** |  |  |  |  |
| BMI, kg/m^2^ | 26.5 (24.1, 29.3) | 27.1 (24.7, 29.3) | 25.9 (22.9, 29.0) | 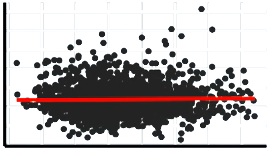 |
| **Be physically active** |  |  |  |  |
| Moderate-vigorous physical activity, min/week | 135 (0, 300) | 135 (15, 345) | 135 (0, 300) | 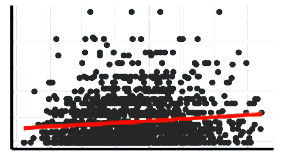 |
| **Eat whole grains, vegetables, fruits and beans** |  |  |  |  |
| Fruits and vegetables, g/day | 422 (273, 596) | 394 (254, 574) | 458 (304, 630) | 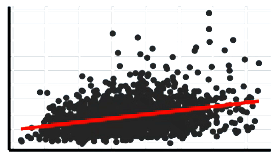 |
| Fiber, g/day | 27.5 (21.7, 35.1) | 28.2 (22.4, 35.5) | 26.8 (20.8, 34.4) | 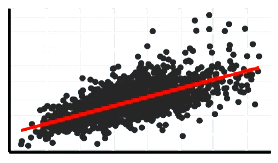 |
| **Limit fast foods and processed foods** |  |  |  |  |
| NOVA-classified aUPFs^3^, E% | 16.4 (11.4, 21.6) | 17.2 (12.4, 22.2) | 14.9 (10.5, 20.1) | 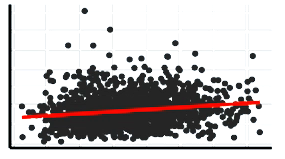 |
| **Limit red and processed meat** |  |  |  |  |
| Red meat, g/day | 70.8 (47.5, 99.0) | 82.4 (56.9, 116.0) | 57.3 (36.4, 81.8) | 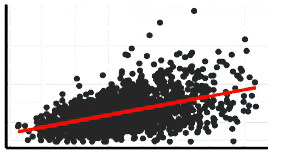 |
| Processed meat, g/day | 47.2 (30.5, 70.1) | 57.7 (37.8, 81.6) | 38.7 (24.3, 55.8) | 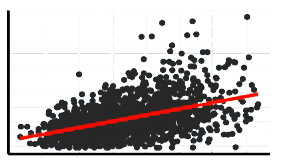 |
| **Limit sugar-sweetened drinks** |  |  |  |  |
| Sugar sweetened drinks, g/day | 0 (0, 42) | 6 (0, 70) | 0 (0, 28) | 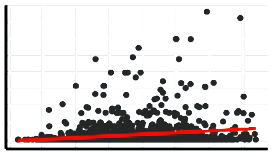 |
| **Limit alcohol** |  |  |  |  |
| Alcohol, g/day | 8.95 (2.2, 19.3) | 12.8 (3.8, 24.9) | 5.4 (1.1, 13.1) | 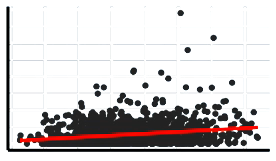 |

*^1^For continuous variables, numbers may vary due to missing information.*

*^2^Scatter plots for the population as a whole with energy intake in kcal/day on the x-axis and the WCRF/AICR Score and the individual diet and lifestyle components on the y-axis.*

*^3^The aUPF variable was created based on the NOVA classification system. Food items already included in other components of the score (e.g. sugar-sweetened drinks and red and processed meats) were left out to avoid double penalization.*

*Abbreviations: AICR; American Institute for Cancer Research, aUPFs; adapted ultra-processed foods, n; number, p; percentile, WCRF; World Cancer Research Fund.*


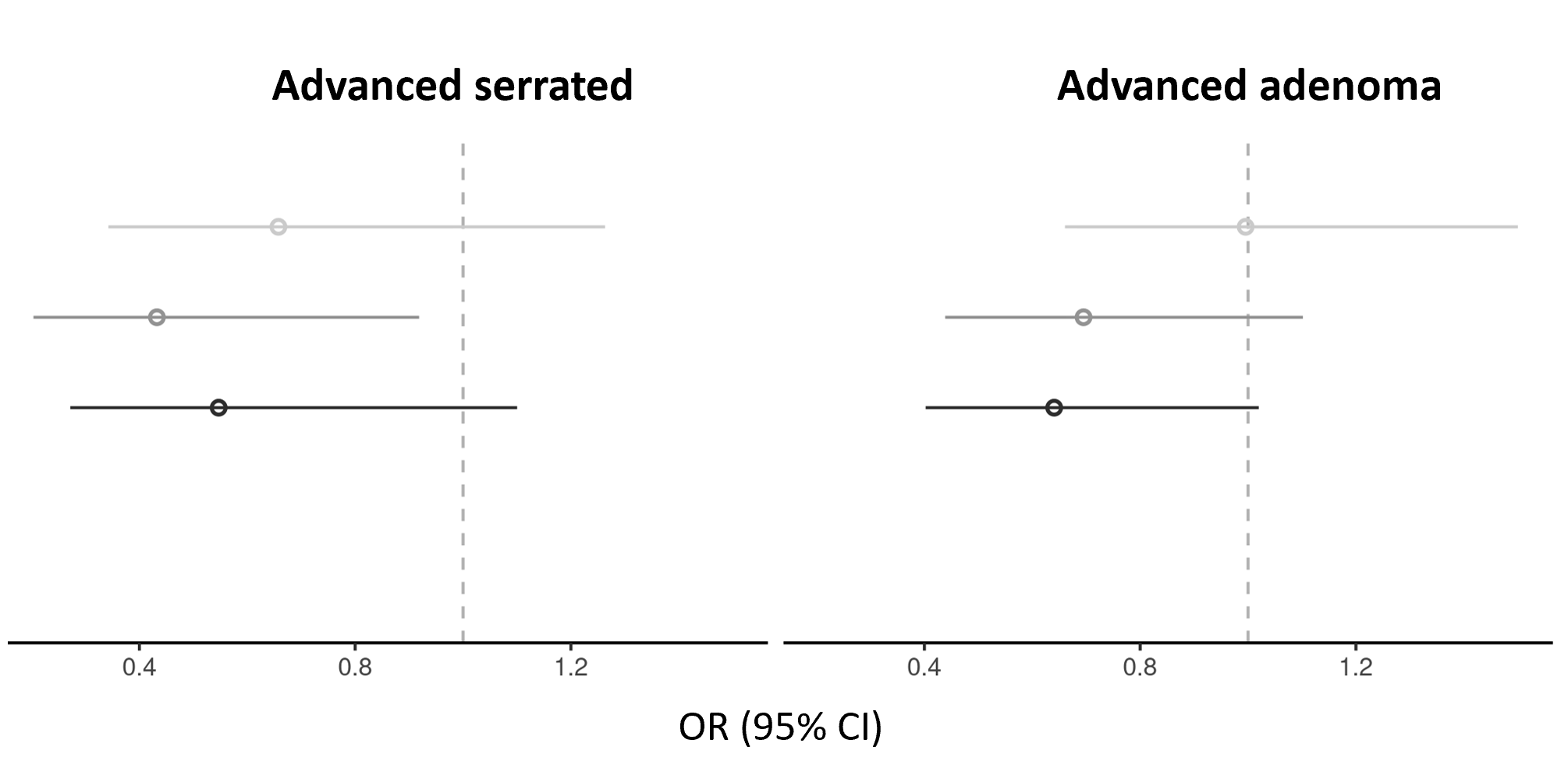


**Supplementary Figure 1**. Odds ratios (ORs) and 95% confidence intervals (CIs) for presence of the two main precursor lesion types advanced adenoma and advanced serrated lesion relative to no adenoma by adherence to the 2018 WCRF/AICR Cancer Prevention Recommendations. Effect estimates are derived from a multinomial logistic regression analysis, including the following clinical groups: No adenoma (n=548), non-advanced adenoma (n=524), advanced serrated lesion (n=74), advanced adenoma (n=238), mixed lesions (n=37) and CRC (n=65). The first quartile (≤ 2.75 points) is treated as the reference category. Analyses are adjusted for age (continuous), sex, energy intake (continuous), smoking status (current smoker, past smoker, non-smoker, missing), education level (primary school, high school, collage/university, missing) and family history of CRC (yes, no, unknown).


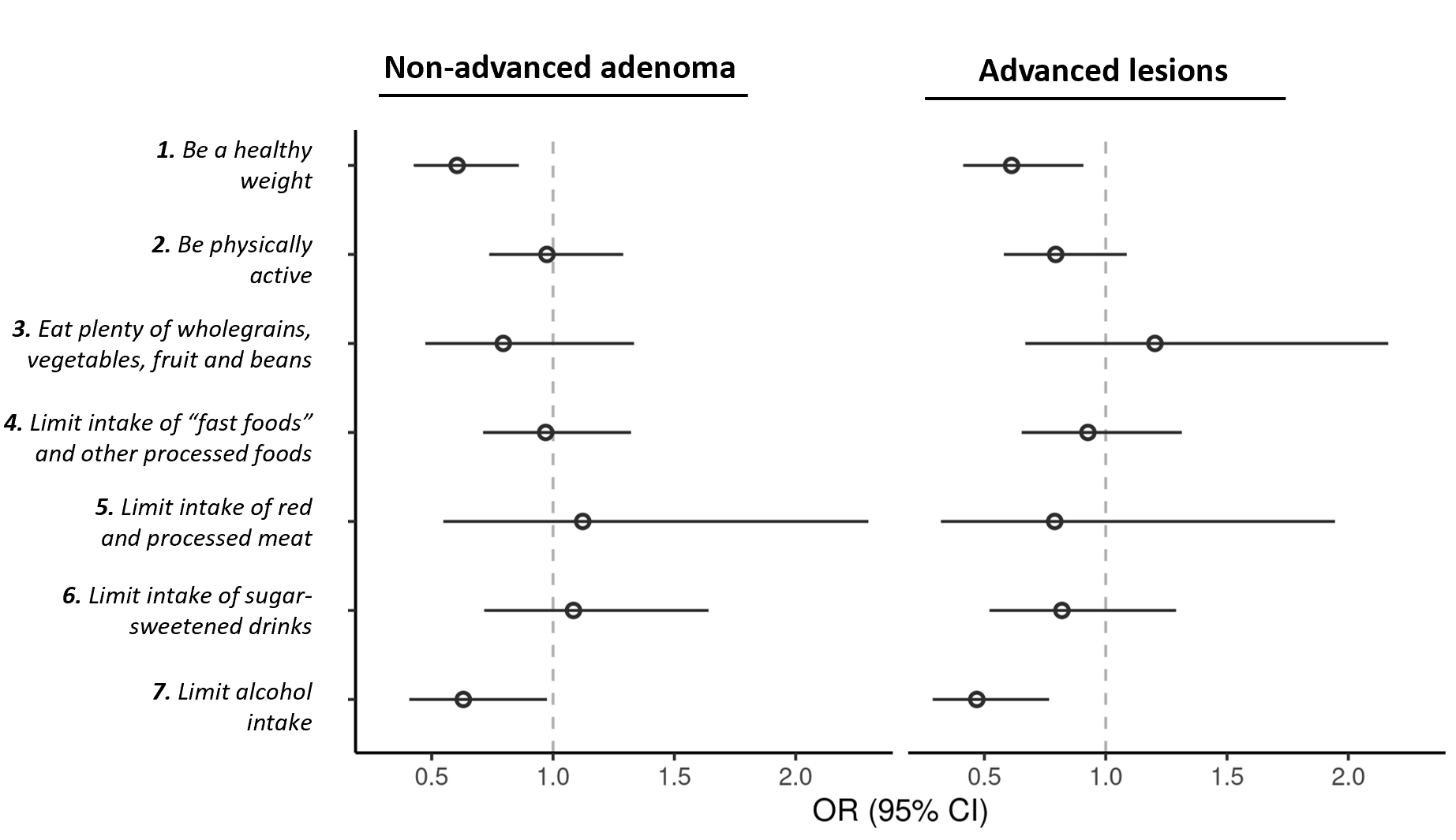


**Supplementary Figure 2**. Odds ratios (ORs) and 95% confidence intervals (CIs) for presence of non-advanced adenoma and advanced lesions relative to no adenoma by adherence to the individual 2018 WCRF/AICR Cancer Prevention Recommendations in the CRCbiome study (n=1,486). Effect estimates are derived from a multinomial logistic regression model also including CRC as outcome category. However, as confidence intervals were extremely wide, effect estimates for this outcome were left out. Effect estimates indicate the probability of lesion detection by one point increase in the score (i.e. going from not adhering to fully adhering). Analyses are adjusted for age (continuous), sex, energy intake (continuous), smoking status (current smoker, past smoker, non-smoker, missing), education level (primary school, high school, collage/university, missing) and family history of CRC (yes, no, unknown).
